# Protection of L1 nerve roots by pre-relieve tension in parallel endplate osteotomy for severe rigid thoracolumbar spine deformity

**DOI:** 10.1186/s12891-020-03288-0

**Published:** 2020-05-15

**Authors:** Hang Liao, Houguang Miao, Peng Xie, Yueyue Wang, Ningdao Li, Guizhou Zheng, Xuedong Li, Shixin Du

**Affiliations:** Department of Orthopedics, The 3rd Affiliated Hospital of Shenzhen University, Shenzhen, 518000 People’s Republic of China

**Keywords:** Thoracolumbar deformity, Nerve roots injury, L1 nerve roots, Parallel endplate osteotomy

## Abstract

**Background:**

This is a retrospective study of the use of parallel endplate osteotomy (PEO) for correction of severe rigid thoracolumbar spine deformity.

**Methods:**

From July 2016 to January 2019, 12 patients with severe rigid thoracolumbar spine deformity underwent PEO on T12 or L1 vertebrae were studied.

**Results:**

Following PEO at T12 or L1, the mean kyphosis and scoliosis correction rates reached 77.0 ± 8.9% and 75.5 ± 8.0%, respectively and the intraoperative estimated blood loss was 1950 ± 1050 mL, and the mean operative time was 6.98 ± 4.02 h. The SF-36 scores of physical function, role-physical, bodily pain, general health, vitality, social function, role-emotional and mental health changed from 63 ± 28, 50 ± 25, 50 ± 30, 34 ± 19, 53 ± 28, 45 ± 30, 30 ± 36 and 54 ± 18 at baseline to 83 ± 18, 69 ± 19, 72 ± 12, 66 ± 21, 75 ± 15, 72 ± 22, 66 ± 34 and 76 ± 12 at 1 year postoperatively, 83 ± 8, 68 ± 32, 83 ± 17, 73 ± 17, 82 ± 18, 76 ± 26, 70 ± 37 and 88 ± 12 at 18 months postoperatively, 86 ± 6, 83 ± 33, 90 ± 16, 81 ± 16, 89 ± 14, 88 ± 25, 83 ± 17 and 94 ± 10 at 24 months postoperatively, respectively (*P* < 0.01). Three patients had symptoms of L1 nerve root injury, as reflected by lower limb weakness and inner thigh numbness on knee extension and hip flexion, which was further confirmed by electromyography.

**Conclusions:**

PEO is easier to operate, and the spinal cord and nerve root are under direct vision and can effectively and safely correct severe rigid thoracolumbar spine deformity with satisfactory clinical results. However, it is important to identify, separate and protect L1 nerve roots during surgery in cases where patients have symptoms of back pain, muscle weakness and leg numbness on the convex side after surgery.

## Background

Severe rigid thoracolumbar spine deformity, a thoracolumbar spinal deformity with a kyphosis or a scoliosis angle of over 80 degrees after bending or traction (flexibility< 25%) [1], is comprised of a variety of spinal deformities, such as untreated adolescent idiopathic scoliosis, spinal congenital malformations, spinal hemivertebra malformations, incomplete segmental malformations and deformity caused by ankylosing spondylitis [2]. As the disease progresses, the thoracolumbar spine becomes ossified and the patient develops chest and back pain with stiffness. Patients with severe rigid thoracolumbar spinal deformity mostly have respiratory restriction, suffocating breathing and limited cardiac output [3].

For severe rigid deformity, spinal osteotomy is recommended [4]. Spinal osteotomy ideally should involve a single surgical approach to decompress nerve roots, correct the deformity and stabilize the spine [5]. Due to the pathological characteristics of severe rigid thoracolumbar spine deformity, osteotomy is extremely challenging for spine surgeons, and is accompanied by a high rate of complications, including neurological lesions, blood loss, infection, pseudarthrosis, fixation failure and loss of correction [6]. Nerve root injury is a common disastrous problem in osteotomy [7]. T12 or L1 are usually taken as the apical vertebra for spinal osteotomy [8]. Because the canalis vertebralis at T12 and L1 contains the spinal cord, nerve roots and medullary cone, spinal osteotomy at T12 or L1 vertebra is more dangerous than at lower levels [9]. As a result, T12 or L1 osteotomy is more likely to injure L1 nerve roots, especially in severely rotated vertebra on the convex side, where L1 nerve roots, unlike T12 nerve roots, could penetrate through the surface of the vertebra, which causes symptoms such as back pain, muscle weakness and leg numbness, thus interfering with daily activities [10]. Therefore, it is necessary to reduce the risk of various complications, so as to effectively protect L1 nerve roots.

Our research group used Mimics medical three-dimensional (3D) reconstruction software to reconstruct 3D digital models of various vertebral bodies, and provide optimal osteotomy and fixed segments for the operation. We utilized parallel endplate osteotomy (PEO) to treat severe rigid thoracolumbar spinal deformity and achieved satisfactory clinical results. The PEO technique is preferable to one-stage osteotomy instead of traction before surgery. In particular, we pay special attention to identification, separation, tension reduction and protection of L1 nerve roots during surgery.

## Methods

### Patients

This retrospective study included 12 patients with severe rigid thoracolumbar spine deformity who underwent PEO at T12 or L1 between July 2016 and January 2019. Deformity was assessed both clinically and radiographically as part of the preoperative planning. Diagnosis of thoracolumbar spine deformity was made by human grid analysis, X-rays after bending or traction, 3D CT and 3D printing models. Patient complaints included serious waist deformity, lower back pain and increased lower limb weakness and leg numbness after activity. All patients underwent a one-stage osteotomy instead of preoperative traction. The inclusion criteria: patients with severe rigid thoracolumbar spine deformity who underwent PEO at T12 or L1**.** The **e**xclusion criteria: patients with spinal cord or nerve root injury or other serious respiratory complications were excluded.

### Assessment of deformity

Using standard Cobb’s method on standing lateral radiograph of the whole spine, the global thoracolumbar kyphosis or scoliosis was measured from the upper endplate of T1 to the lower endplate of L5. Severe rigid spinal deformity was defined as having curve angles more than 80°, with flexibility less than 25% by X-rays after bending or traction [1], exclusion patients with spinal cord or nerve root injury or have other serious respiratory complications. Radiographic parameters were evaluated, and clinical records were reviewed. The osteotomy location was usually chosen as the vertebra that contributed most to the deformity, according to the apex of the deformity. Such a measurement not only represents the severity of the overall deformity, but also determines the amount of correction to be achieved by spinal osteotomy.

### The surgical procedure

The patient was placed prone over the table, which was in a flat position throughout the operation. The location of the upper and lower end vertebrae was determined by mobile C-arm X-ray imaging before surgery. The vertebral column from the upper to the lower end vertebrae was prepared and draped. A straight vertical midline incision was made over the spinous processes and was extended to the upper and lower end vertebrae. If the deformed vertebra had a rotation, an incision was made on the convex side, deviating approximately 1–2 cm from the centerline. The paraspinal muscles were dissected subperiosteally from the spinous processes and laminae, and then retracted laterally. After a careful dissection of the area around the facet joints, a large spinal retractor was applied. By spreading the spinal retractor and detachment of muscles around the facet joints, a wider exposure was obtained. The spinal retractor could reduce soft tissue damage and infection, and avoid long-term traction. An intraoperative radiograph with guide pins was obtained for accurate localization of the deformity and determination of the level and area for osteotomy. Pedicle screws were then inserted into the segments from the upper end vertebra to the lower end vertebra using a free-hand technique at all levels planned prior to surgery. The osteotomy site was usually chosen as the vertebra that contributed most to the deformity, according to the apex of the deformity.

Usually the spine was stabilized with a short bent rod in situ adjacent to the resected area to avoid coronal and sagittal plane translation during the reduction maneuver. The first unilateral rod was temporarily fixed on the concavity side bend to maintain spinal stability after PEO. A complete laminectomy and facetectomy was performed to expose the spinal cord. Usually, the spinal cord was located in the concave curve side, sometimes slightly located in the convex curve side. If the spinal cord was located in the convex curve side, greater caution should be exercised because of neurological complications due to high tension of the spinal cord. In some cases, the spinal cord was as tight as a cord with the diameter of only one-third of a normal spinal cord. Any slight maneuver would make the action potentials to decline sharply by over 50%, or even disappear. Timely identification and prompt intervention must be performed, including enlarging the resected area to reduce the abrupt turning tendency of the spinal cord.

In our experience, we removed two levels of nerve roots of the thoracolumbar spinal cord for PEO, if necessary. These procedures allow circum-spinal decompression of the spinal cord. One of the critical pitfalls in this step was the careless mistake of pulling out the preserved spinal nerve roots at the corresponding level. This was quite dangerous for the spinal cord, which was already compressed at the apex of the angular kyphotic deformity, because pulling out the nerve root increased the pressure of the spinal cord at the apex. When the initial PEO was carried out, we did not pay special attention to the L1 nerve roots. Due to position variation, we made the careless mistake of regarding L1 as T12 nerve roots and damaged L1, which was confirmed by lower limb EMG after surgery. Thereafter, we attempted to use a nerve stripper to separate and release the tension of the L1 nerve roots under somatosensory-evoked potential (SEP) and motor-evoked potential (MEP) monitoring, ultimately leaving the L1 nerve roots slack and floating in the gap.

**Fig. 1:**
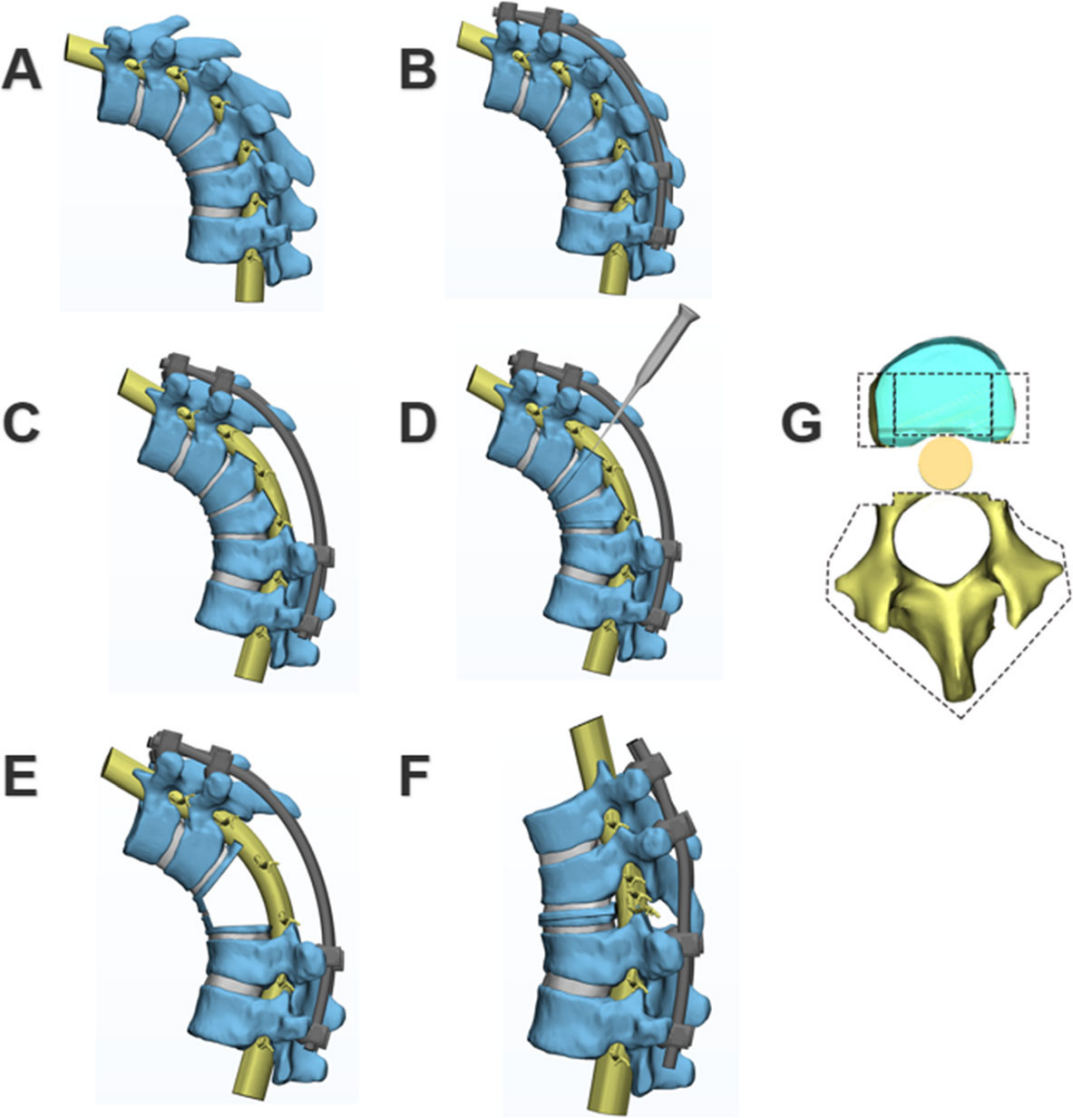
**a**, **b**, **c**, **d**, **e** and **f** The three-dimensional demonstration of parallel endplate osteotomy (PEO). **g** Osteotomy range includes pedicle of the vertebral arch, 2/3 posterior vertebra, bilateral walls of the vertebra and the posterior wall of the vertebra

For PEO, the pedicle of the vertebral arch, 2/3 of the posterior vertebra, the bilateral walls of the vertebra and the posterior wall of the vertebra (5 mm to endplate) were carefully removed using an osteotome, curette, rongeur and ultrasonic osteotome (Fig. 1). The parallel endplate osteotomy area had two situations: a single vertebral osteotomy if the angle of the curve was less than 90°, or a multiple vertebral osteotomy if the angle of the curve was greater than 90°. If the spinal cord was compressed at the apex of the angular deformity, the lesion compressing the spinal cord was also drilled out using an L-shaped bone separator under direct vision from the lateral direction. A thin stripper was used to confirm whether the soft tissue, such as the posterior longitudinal ligament, attached to the dural sac was soft enough.

The osteotomy was performed carefully to avoid over-penetration of the anterior vertebral body cortex or anterior intervertebral disc for the purpose of preventing injury to the major vessels in front of the vertebral body. Then, we inserted another precontoured correction rod on the convex side to exchange the rods, 30 degrees per correction. It was important in this step to keep an adequate compression force on the concave rod while its adjunct screws on the cephalic side were slightly released until the concave rod and screws were tightened one by one. In situ rod bending on the concave side should never be performed because it is very dangerous procedure to the naked spinal cord, and applying too much torsion to the pedicle screws could easily cause screw loosening and rod bender stick out and injure the spinal cord. After repeated compressions and shuttling segmental transient rods, finally, we placed the terminal fixation rods after main correction was achieved. Then, segmental derotation, compression, and distraction on the secondary curves were performed to achieve final correction. During the entire correction procedure, the dural sac was closely observed to avoid migration in any direction, and tension of the spinal cord was assessed by observation and frequent palpation. Adequate and quick adjustments were needed to ensure that spinal cord tension did not exceed the initial state under distraction, and to prevent excessive kinking of the dural sac after spinal shortening. Kawahara et al. confirmed that the spine that was shortened within one-third of the height of the vertebrae would not lead to a functional change of the spinal cord [11]. During osteotomy, we had a maximum spinal shortening of 5 cm, there were no warning signs by SEP and MEP. After completion of resection and deformity correction, any residual gap was filled with resected vertebral body bone morsels. Finally, we checked that the spinal cord was thoroughly smooth. We gave patients an autologous blood transfusion that was recycled, or allogeneic blood transfusion if the volume of bleeding was high.

### Intraoperative monitoring technique and postoperative follow-up

We monitored the somatosensory-evoked potential (SEP) and motor-evoked potential (MEP) to effectively monitor the spinal cord and nerve roots under the supervision of an experienced neurophysiologic physician throughout the PEO procedure, and an additional wake-up test was performed after finishing the correction step at the end of the surgery to ensure the neurological status. Intraoperative and postoperative complications were recorded. For patients with L1 nerve roots injury, continuous electromyography (EMG) monitoring was necessary. In an attempt to validate these patients’ clinical outcome, 12 patients were also asked to respond to the SF-36 quality of life questionnaire via telephone interview after a year of follow-up.

## Results

In total, 12 severe rigid thoracolumbarspine deformity patients received a PEO at T12 or L1. The three-dimensional model provided accurate diagnostic and better surgical options. The kyphosis and scoliosis correction rates reached 77.0 ± 8.9% and 75.5 ± 8.0%, respectively. The mean intraoperative estimated blood loss was 1950 ± 1050 mL and the mean operative time was 6.98 ± 4.02 h. Osteotomies were all performed at T12 or L1. Three patients had typical symptoms of L1 nerve root injury. Specifically, the lower limb exhibited weakness on knee extension and hip flexion, and the inner thigh felt numb (Table 1). Tables 2 and 3 shows SF-36 scores of the patients at baseline, 1 year, 18 months and 24 months postoperatively, respectively. The SF-36 scores of physical function, role-physical, bodily pain, general health, vitality, social function, role-emotional and mental health changed from 63 ± 28, 50 ± 25, 50 ± 30, 34 ± 19, 53 ± 28, 45 ± 30, 30 ± 36 and 54 ± 18 at baseline to 83 ± 18, 69 ± 19, 72 ± 12, 66 ± 21, 75 ± 15, 72 ± 22, 66 ± 34 and 76 ± 12 at 1 year postoperatively, 83 ± 8, 68 ± 32, 83 ± 17, 73 ± 17, 82 ± 18, 76 ± 26, 70 ± 37 and 88 ± 12 at 18 months postoperatively, 86 ± 6, 83 ± 33, 90 ± 16, 81 ± 16, 89 ± 14, 88 ± 25, 83 ± 17 and 94 ± 10 at 24 months postoperatively, respectively (*P* < 0.01, Student’s t test), indicating that the quality of life of the patients improved significantly after PEO.

**Table 1:** Summarized data of patients

Patient No.	Age/Sex	Etiology	Kyphosis Cobb (degrees) Pre-op Post-op	Scoliosis Cobb (degrees) Pre-op Post-op	Osteotomy segments	Upper and lower end vertebra	Bleeding (mL)	L1 nerve roots injury	Operative time (hours)
1	33/F	Idiopathic	85 12	67 15	T11/T12	T8-L4	1800	YES	5.6
2	21/M	Idiopathic	90 25	130 40	T12/L1	T5-L5	3000	YES	11
3	25/F	Congenital	112 20	98 18	T12/L1	T8-L5	1500	NO	5.8
4	8/M	Tuberculosis	98 16	–	T10/T11/T12	T5-L4	1000	YES	6
5	22/M	Idiopathic	102 32	118 35	L1/L2	T8-S1	3000	NO	5.5
6	10/F	Congenital	90 22	78 16	T11/T12	T6-L3	1500	NO	5
7	15/M	Idiopathic	108 24	131 35	T9/T11/T12	T4-L5	2600	NO	9
8	24/F	Congenital	100 15	85 14	T12/L1	T9-L5	1800	NO	6.3
9	16/M	Congenital	148 44	–	T12/L1	T5-L4	2000	NO	10
10	20/F	Congenital	166 46	–	T10/T11/T12	T5-L5	1700	NO	7
11	38/F	Idiopathic	85 25	63 15	T12/L1	T9-L4	2500	NO	5.5
12	32/M	Congenital	100 20	54 17	T12	T8-L4	1000	NO	7

**Table 2:** Comparison of SF-36 scores before and 1 year after surgery

Patient No.	Physical Function Pre Post	Role-Physical Pre Post	Bodily Pain Pre Post	General Health Pre Post	Vitality Pre Post	Sociall Function Pre Post	Role-Emotional Pre Post	Mental Health Pre Post
1 (33/F)	80 85	75 75	62 84	30 45	65 80	50 75	33 66	52 76
2 (22/M)	40 80	25 50	41 62	20 70	55 70	50 75	0 33	52 72
3 (25/F)	75 85	50 75	51 62	25 55	50 75	75 75	66,100	40 84
4 (33/F)	80 90	75 75	74 80	45 65	75 85	50 75	66,100	72 84
5 (21/M)	90 85	75 75	74 80	45 65	80 90	25 75	33 66	68 80
6 (10/F)	50 75	25 75	22 62	20 70	40 65	25 50	33 66	48 76
7 (15/M)	35 65	25 50	20 62	25 65	25 60	38 63	0 33	52 68
8 (24/F)	45 85	25 50	22 74	15 60	45 70	50 75	0 33	36 64
9 (16/M)	55 75	50 75	32 74	40 75	25 65	50 63	0 66	48 72
10 (20/F)	50 85	50 75	40 82	50 85	40 75	50 88	33 66	56 84
11 (38/F)	85 90	75 75	51 62	45 70	65 80	25 75	33 66	52 76
12 (32/M)	75 90	50 75	74 84	45 65	75 85	50 75	66,100	72 80

**Table 3:** The follow-up SF-36 scores 18 (a) and 24 (b) months after surgery

Patient No.	Physical Function a b	Role-Physical a b	Bodily Pain a b	General Health a b	Vitality a b	Sociall Function a b	Role-Emotional a b	Mental Health a b
1 (33/F)	85 90	75 75	84 84	60 70	85 90	75 75	66 66	84 88
2 (22/M)	80 80	50 50	74 74	75 85	75 75	75,100	33 66	76 84
3 (25/F)	75 80	50 75	74 84	65 70	80 85	75 75	66,100	92,100
4 (33/F)	90 90	75,100	100,100	75 90	90 95	75 88	66,100	88,100
5 (21/M)	80 90	100,100	100,100	70 75	100,100	100,100	100,100	100,100
6 (10/F)	80 85	50 75	74 84	75 80	75 85	50 63	66 66	80 84
7 (15/M)	85 80	75 75	74 84	70 85	70 80	63 75	33 66	76 84
8 (24/F)	80 85	50 75	84 84	65 65	85 90	75,100	66 66	84,100
9 (16/M)	80 90	75,100	84,100	80 90	75 90	75,100	100,100	100,100
10 (20/F)	90 90	75,100	84,100	90 95	85,100	100,100	100,100	100,100
11 (38/F)	–	–	–	–	–	–	–	–
12 (32/M)	–	–	–	–	–	–	–	–

However, although the clinical effect of the PEO technique was obvious, complications were unavoidable. L1 nerve roots injury occurred in 3 patients, with abnormal SEP and MEP waveforms during the operation that was confirmed by lower limb EMG after surgery. The symptoms of L1 nerve root injury were significantly improved by pharmacotherapy with mannitol and methylprednisolone and nutritional neurotherapy. We could see that L1 nerve root function was obviously improved by continuously monitoring EMG of both lower limbs. Meanwhile, 1 case developed hemopneumothorax, which was effectively repaired without any leakage, and a closed thoracic drainage tube was placed post operation. One patient experienced paralytic ileus which improved after gastric decompression, promoting intestinal motility and symptomatic medical treatment. At 1 year follow up, we did not observe any other complications, such as dura laceration, superficial infection, nonunion/rod breakage, distal screw loosening and adjacent segment kyphosis (Table 4).

**Table 4:** Complications in the study patients

Complication	Patients (*n* = 12)
Dura laceration	0
L1 nerve root injury	3
Paralytic ileus	1
Hemothorax	1
Superficial infection	0
Nonunion/rod broken	0
Distal screw loosening	0
Adjacent segment kyphosis	0

### Data analysis

A Student’s t test was used to evaluate the differences after surgery. Statistical significance was set at a value of *P* < 0.01. Data are displayed as mean ± SD unless otherwise indicated.

### Case 1

**Fig. 2:**
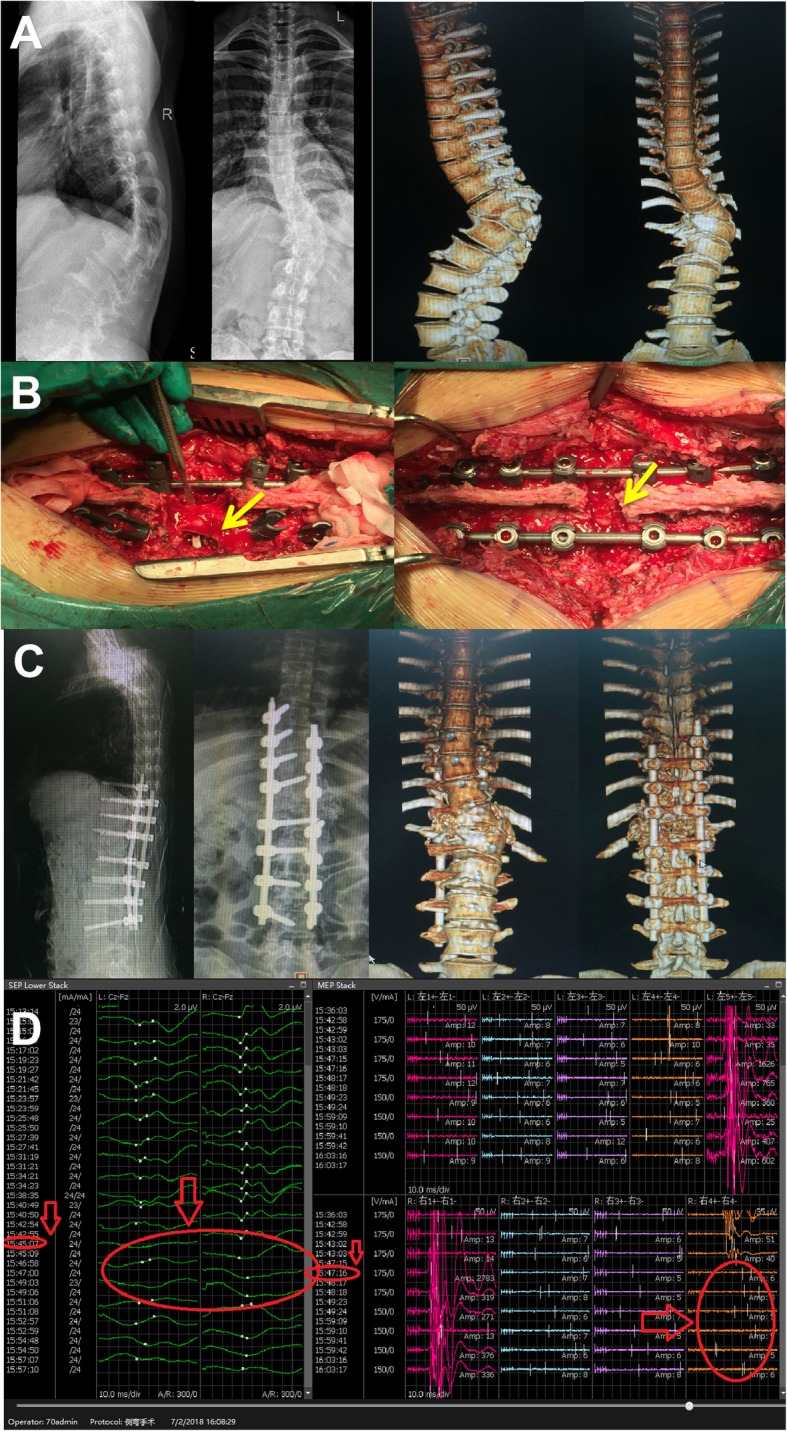
Case 1, female, 33 years of age, severe rigid thoracolumbar deformity. **a** Preoperative diagnosis was kyphosis Cobb 85° and scoliosis Cobb 67° by X-ray after bending and three-dimensional CT imaging. **b** Intraoperative osteotomy, pedicle screw fixation and correction. Yellow arrow represents injury in L1 nerve root on the convex side of scoliosis. **c** Postoperative kyphosis and scoliosis Cobb angle was corrected to 12° and 15°, which show in X-ray and three-dimensional CT imaging. **d** Abnormal waveforms occurred in SEP and MEP of the left lower limb during surgery. Red arrow represents abnormal signal

A 33-year old housewife had waist deformity for 18 years that worsened over the last year. The spine deformity was serious. In the flexion test, the left side of the waist was raised 10 cm, the muscle strength of both lower limbs was grade V, and the patient had normal sensation. The preoperative diagnosis was severe rigid thoracolumbar deformity; kyphosis Cobb 85° and scoliosis Cobb 67° was determined by X-ray after bending (Fig. 2a). Osteotomy was performed at T11 and T12, and the upper and lower end vertebrae were T8 and L4 (Fig. 2b). Due to position variation, we mistakenly identified L1 as the T12 nerve root, and damaged the L1 nerve root on the convex side of the side bend with abnormal waveforms by SEP and MEP during the operation (Fig. 2d). Despite postoperative kyphosis and scoliosis correction to Cobb 12° and 15° (Fig. 2c), knee extension and hip flexion of the left lower limb were weak (grade III), and the inner thigh was numb. We reconfirmed L1 nerve root injury by lower limb EMG after surgery. After pharmacotherapy with mannitol and methylprednisolone and nutritional neurotherapy, the muscle strength of the left lower limb recovered to grade IV, and numbness was relieved before the patient was discharged from the hospital. At 1 year follow-up after surgery, the patient still had left lower limb weakness, which had an impact on daily life.

### Case 2

**Fig. 3:**
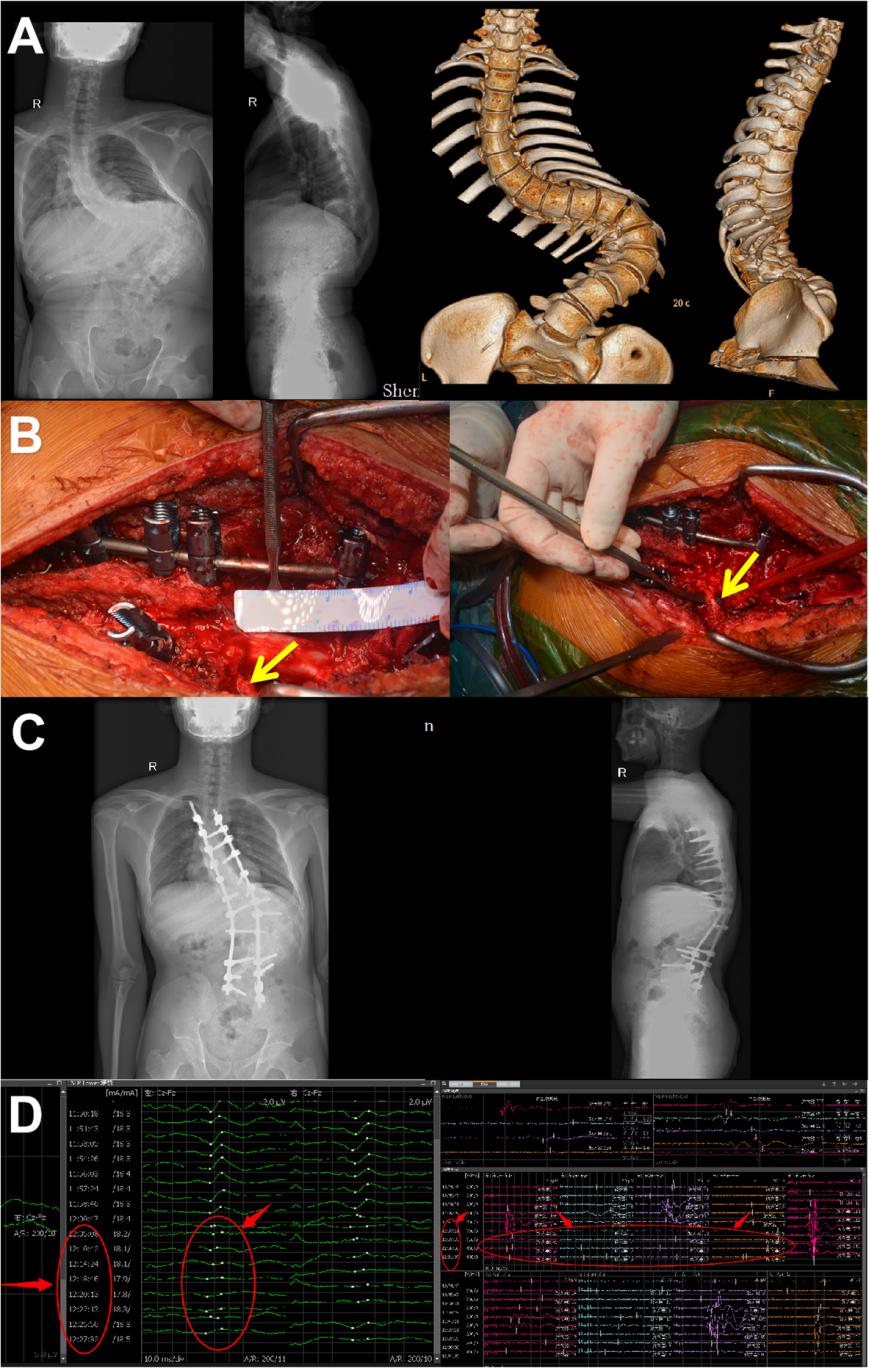
Case 2, male, 21 years old, severe rigid thoracolumbar deformity. **a** Preoperative diagnosis was kyphosis Cobb 90° and scoliosis Cobb 130° by X-ray after bending and three-dimensional CT imaging. **b** Intraoperative osteotomy, pedicle screw fixation and correction. Yellow arrow represents injury in the L1 nerve root on the convex side. **c** Postoperative kyphosis and scoliosis Cobb angle was corrected to 25° and 40°, as shown in X-ray images. **d** Abnormal waveforms occurred in SEP and MEP of the left lower limb during surgery. Red arrow represents abnormal signal

A 21-year old delivery man had waist deformity for 10 years that was worsened over the past 4 years. In the flexion test, the left waist was raised 8 cm, the muscle strength of both lower limbs was grade V, and there was no numbness. The preoperative diagnosis was severe rigid thoracolumbar deformity; kyphosis Cobb 90° and scoliosis Cobb 130° was determined by X-rays after bending (Fig. 3a). Osteotomy was performed at T12 and L1, and the upper and lower end vertebrae were T5 and L5 (Fig. 3b). Due to high tension, we mistakenly damaged the L1 nerve root on the convex side with abnormal waveforms by SEP and MEP during the operation (Fig. 3d). Despite postoperative kyphosis and scoliosis correction to Cobb 25° and 40° (Fig. 3c), knee extension and hip flexion of the left lower limb were grade II, and the inner thigh was numb. After surgery, L1 nerve root injury was confirmed by EMG. After a period of therapy, the muscle strength of the left lower limb recovered to grade IV, and numbness was markedly reduced before the patient was discharged from the hospital. At 1 year follow-up, the patient could not go up the stairs smoothly, which had an impact on his work.

### Case 3

**Fig. 4:**
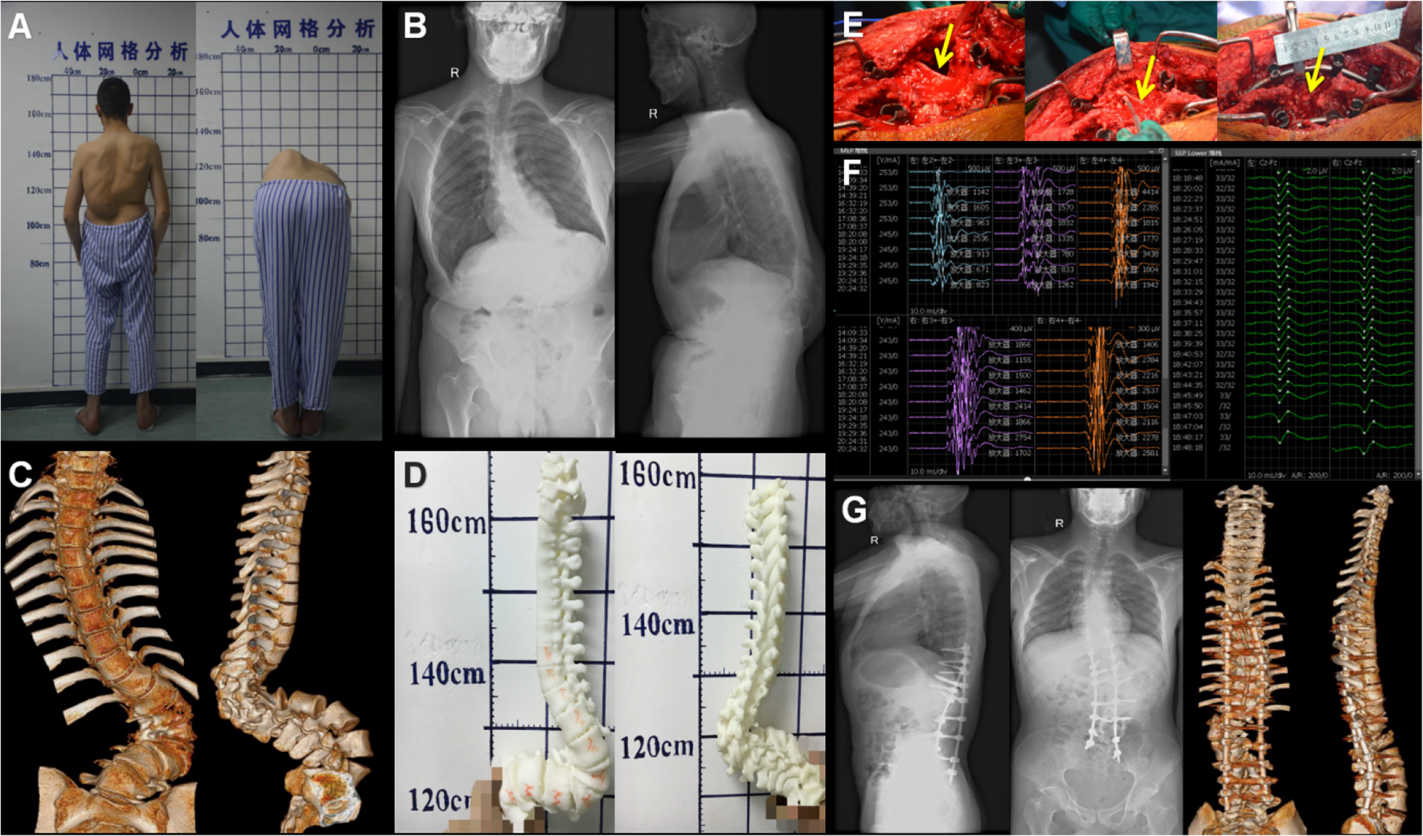
Case 3, male, 22 years old, severe rigid thoracolumbar deformity. **a**, **b**, **c** and **d** Preoperative diagnosis was kyphosis Cobb angle 102° and scoliosis Cobb angel 118° by profile, X-ray after bending, three-dimensional CT and printing model imaging. **e** Intraoperative osteotomy, pedicle screws fixation and correction. Yellow arrow represents the intact and slack L1 nerve root. **f** Normal waveforms occurred in SEP and MEP during surgery. **g** Postoperative kyphosis and scoliosis Cobb angle was corrected to 32° and 35°, as shown in X-ray and three-dimensional CT images

A 22-year old male had waist deformity for 8 years that was worsened over the preceding 2 years. In the flexion test, the left side of the waist was raised 14 cm, and the muscle strength of both lower limbs was grade V, indicating serious spine deformity (Fig. 4a). Preoperative diagnosis was severe rigid thoracolumbar kyphosis, with a kyphosis Cobb angle of 102° and a scoliosis Cobb angle of 118° as judged by X-ray after bending (Fig. 4b, c and d). Osteotomy was performed at L1 and L2, and the upper and lower end vertebrae were T8 and S1. During the operation, we observed that the L1 nerve roots on the convex side of the scoliosis were pulling tension and easily damaged (Fig. 4e). We tried to use nerve strippers to separate and protect the L1 nerve root while maintaining normal waveforms by SEP and MEP during the operation (Fig. 4f). The L1 nerve roots were slack and floating in the gap (Fig. 4e). Kyphosis and scoliosis were corrected to Cobb 32° and 35°, respectively, postoperatively (Fig. 4g) and the patient had no symptoms of nerve root injury.

## Discussion

Surgical intervention is the only effective method for correction of severe rigid thoracolumbar spine deformity, and can restore the sagittal balance of the spine and the horizontal gaze, improve respiratory function, relieve pain and enhance quality of life [12]. Current surgical methods include pedicular subtraction osteotomy (PSO) and vertebral column resection (VCR) [12]. PSO is described as a vertebral wedge osteotomy for correction of spine deformity. It has the advantage of obtaining correction through three columns, from the posterior approach, without lengthening the anterior column, thereby maximizing healing potential while avoiding stretching the major vessels and viscera anterior to the spine [13]. The mean estimated blood loss in PSO procedures has been reported in the literature to be from 1400 mL to 2600 mL [14], and the mean operative time was 9.1 h [15]. The VCR technique can achieve a 360° osteotomy of the entire spine and have yielded satisfactory results [16]; in this technique, the intraoperative estimated blood loss is generally around 3000–5000 mL, and the mean operative time is 9.3 h [17]. For spine deformity, PSO and VCR are classic techniques and can achieve satisfactory results. Meanwhile, the PEO technique provides a new alternative treatment for spinal deformities, with an estimated blood loss of 1950 ± 1050 mL and a mean operative time of 6.98 ± 4.02 h. PEO includes the pedicle of the vertebral arch, 2/3 of the posterior vertebra, the bilateral walls of the vertebra and the posterior wall of the vertebra (5 mm to the endplate), which does not require through pedicular osteotomy (like PSO) and resection of the intervertebral disc above and below the osteotomy site (like VCR). Moreover, the endplate as a mark in PEO is easy to identify which has a large operating space, and is especially suitable for pedicle deformity or agenesis which is unrecognizable, which is easier to operate for orthopedic surgeons. With bone-bone fusion, we can achieve a higher spinal fusion rate and better spine stability, in order to reduce the risk of rod breakage. Despite the low risk of rod breakage, at 1 year follow-up, we still have a case of rod breakage who underwent PEO not at T12 or L1. Due to pregnancy after surgery, the increased force on the spine may lead to rod breakage.

In addition, Valone et al. observed that lumbar nerve root weakness or injury can be variously attributed to operative manipulation or decompression and occurs in up to 30% of spinal deformity cases [18]. Compressive injury may also occur during manipulation of the spinal column, especially after a 3-column osteotomy, whereby osseous or ligamentous structures come to rest on the nerve roots in proximity to the osteotomy [19]. Over-pulling nerve roots often causes degeneration, leading to lower limb weakness and numbness. Moreover, nerve root tension often leads to position variation. As a result, the slim nerve root is often located on the lateral side of the scoliosis vertebrae, where it is easily damaged. Sometimes, L1 nerve roots are more likely to appear in the position of T12. In our experience, retention of the T12 nerve root is not required, due to its relatively low influence on lower limb function. However, we once mistook L1 for the T12 nerve root and damaged it. After osteotomy, the L1 nerve roots are easily pinched in the osteotomy space. In general, for T12 or L1 osteotomy, predicting and preventing L1 nerve root injury are paramount in improving postoperative health-related quality of life and function.

Therefore, we reconstruct a 3D digital model of all vertebral bodies to simulate the spinal cord changes in severe rigid spinal deformity after osteotomy correction. It can provide more reasonable solutions for ideal correction degrees, thoracic reshaping, estimation of the spinal cord, post-somatic correction and nerve root safety. Severe rigid spinal deformity still follows the traditional method of preoperative traction. Traction as a form of spinal orthopedics has been widely used, and plays an important auxiliary role in preoperative correction and second-stage osteotomy [20]. Meanwhile, traditional traction can cause additional pain to the patient and is more prone to serious complications, such as cranial nerve injury, cervical spondylosis, loosening and infection [21]. Our team used the PEO technique for one-stage osteotomy, which does not need traction before surgery, dramatically reducing patient suffering, treatment costs and various complications.

In some cases, the L1 nerve roots are pulled tight, causing position variation, and can adhere to surrounding tissues. L1 nerve root injury often causes lower limb weakness and numbness. As a result, we used a nerve stripper to separate, pre-relieve tension and protect L1 nerve roots, while monitoring the process by the SEP and MEP, in order to reduce the risk of nerve root damage. The osteotomy angle can reach 110°-140°, basically satisfying any angle requirements for correction of spinal deformity. The correction rate can reach 70–86%, which is better than the traditional correction rate of 55–60% [22]. During osteotomy, we had a maximum spinal shortening of 5 cm, but we did not worry about the excessive ruga of the dural sac. Because there were no monitoring warning signs by SEP and MEP, after osteotomy, the postoperative bone-bone fusion rate was significantly improved with intervertebral bone graft. Short-term complications after surgery, such as L1 nerve root injury, paralytic ileus and hemithorax all improved or were cured by appropriate treatment. At 1 year follow-up after surgery, it is obvious from the SF-36 scores that all patients achieved better clinical results and with no other complications. In addition, we are aware of the importance of protecting L1 nerve roots.

## Conclusions

We performed parallel endplate osteotomy (PEO) directly on the vertebral body (5 mm to the endplate), and 1/3 of the vertebral body as a mechanical orthopedic hinge. The endplate as a mark in PEO is easy to identify which has a large operating space, and is especially suitable for pedicle deformity or agenesis which is unrecognizable, with bone-bone fusion, which is easier to operate, spinal cord and nerve root under direct vision and can effectively and safely correct severe rigid thoracolumbar spine deformity with better clinical results. Nerve roots injury is a commonly disastrous problem in osteotomy. As a result, due to position variation, T12 or L1 osteotomy is more likely to damage the L1 nerve roots, which are not easy to identify, especially in the severely rotated vertebra on the convex side, and, unlike T12 nerve roots, are usually approached through the surface of the vertebra which causes symptoms such as back pain, muscle weakness and leg numbness, thus interfering with daily activities. Therefore, it is necessary to reduce the risk of various complications, so as to effectively protect L1 nerve roots.


**Additional file 1.**



## Data Availability

All data generated or analysed during this study are included in this published article and its supplementary information files.

## Abbreviations

PEO: Parallel endplate osteotomy
SEP: Somatosensory-evoked potential
MEP: Motor-evoked potential
EMG: Electromyography
PSO: Pedicular subtraction osteotomy
VCR: Vertebral column resection
